# Translating evidence into policy for cardiovascular disease control in India

**DOI:** 10.1186/1478-4505-9-8

**Published:** 2011-02-09

**Authors:** Rajeev Gupta, Soneil Guptha, Rajnish Joshi, Denis Xavier

**Affiliations:** 1 Fortis Escorts Hospital, Jaipur 302017, India; 2 Mahatma Gandhi Institute of Medical Sciences, Wardha 442102, India; 3 St John's Medical College, Bangalore 560038, India

## Abstract

Cardiovascular diseases (CVD) are leading causes of premature mortality in India. Evidence from developed countries shows that mortality from these can be substantially prevented using population-wide and individual-based strategies. Policy initiatives for control of CVD in India have been suggested but evidence of efficacy has emerged only recently. These initiatives can have immediate impact in reducing morbidity and mortality. Of the prevention strategies, primordial involve improvement in socioeconomic status and literacy, adequate healthcare financing and public health insurance, effective national CVD control programme, smoking control policies, legislative control of saturated fats, trans fats, salt and alcohol, and development of facilities for increasing physical activity through better urban planning and school-based and worksite interventions. Primary prevention entails change in medical educational curriculum and improved healthcare delivery for control of CVD risk factors-smoking, hypertension, dyslipidemia and diabetes. Secondary prevention involves creation of facilities and human resources for optimum acute CVD care and secondary prevention. There is need to integrate various policy makers, develop effective policies and modify healthcare systems for effective delivery of CVD preventive care.

## Introduction

It is now established that non-communicable diseases especially cardiovascular diseases (CVD) are major causes of death and disability in low income countries including India [1]. In India the latest Registrar General of India report confirms that circulatory diseases [CVD, coronary heart disease (CHD) and stroke] are the largest cause of deaths. This is observed in all regions of the country, in men and women (Table 1) [2]. Prevalence of CVD and its risk factors is rapidly increasing [3] and it causes major burden on healthcare systems [4]. Although policy initiatives for prevention and control of CVD and other chronic diseases in India have been proposed earlier [4], some evidence for their efficacy is now emerging within the country. This is in addition to international evidence of efficacy of these measures [5]. This essay summarises current data on epidemiology of CVD in India and suggests evidence-based policy interventions for their prevention and control.

**Table 1:** Top ten causes of deaths in India classified according to areas of residence and gender

Rank	India (all age groups)	Economically backward states	Economically advanced states	Rural populations	Urban populations	Men	Women	Middle-age (25-69 years)
1	Cardiovascular	Cardiovascular	Cardiovascular	Cardiovascular	Cardiovascular	Cardiovascular	Cardiovascular	Cardiovascular
2	COPD, asthma	Diarrhoeas	COPD, asthma	COPD, asthma	Cancers	COPD, asthma	Diarrhoeas	COPD, asthma
3	Diarrhoea	Respiratory infections	Cancers	Diarrhoeas	COPD, asthma	Tuberculosis	COPD, asthma	Tuberculosis
4	Perinatal	COPD, asthma	Senility	Perinatal	Tuberculosis	Diarrhoeas	Respiratory infections	Cancers
5	Respiratory infections	Perinatal	Diarrhoeas	Respiratory infections	Senility	Perinatal	Senility	Ill-defined
6	Tuberculosis	Tuberculosis	Tuberculosis	Tuberculosis	Diarrhoeas	Cancers	Perinatal	Digestive diseases
7	Cancers	Other infections	Injuries	Cancers	Injuries	Respiratory infections	Cancers	Diarrhoeas
8	Senility	Ill defined	Perinatal	Senility	Ill-defined	Injuries	Ill defined	Injuries
9	Injuries	Injuries	Ill defined	Injuries	Digestive	Ill defined	Tuberculosis	Suicides
10	Ill defined	Malaria	Respiratory infections	Ill defined	Respiratory infections	Senility	Injuries	Malaria

Adapted from Registrar General of India report (2009)^2^

## Cardiovascular diseases in India

World Health Organization (WHO) reports that non-communicable chronic diseases (NCDs) are responsible for about 70% of all worldwide deaths [5]. In India mortality data from Registrar General of India prior to 1998 were obtained from predominantly rural populations where vital registration varied from 5-15% [6]. The Million Death Study collected mortality statistics from all the Indian states using country-wide Sample Registration System units [2]. Causes of deaths in more than 113,000 subjects from 1.1 million homes were analysed using a validated verbal autopsy instrument as reported earlier [7]. CVD were the largest causes of deaths in males (20.3%) as well as females (16.9%) and led to 1.7-2.0 million deaths annually. Regional studies have also reported that CVD is the leading cause of deaths in urban [8] as well as rural [9] populations. WHO has predicted that from years 2000 to 2020 DALYs lost from CHD in India shall double in both men and women from the current 7.7 and 5.5 million respectively [3]. Prevalence studies report that CHD diagnosed using history and ECG changes have trebled in both urban and rural adults from early 1960s and current prevalence rates are 10-12% in urban and 4-5% in rural adults [3]. Stroke is also increasing in India [3] and incidence registries using population-based surveillance have reported that annual incidence of stroke varies from 100-150/100,000 population in urban locations with greater incidence in rural regions [10-13]. These studies provide only limited information and there is need for properly designed prospective studies to correctly identify trends.

The increase in CHD and stroke in India is largely an urban phenomenon and only recently a rapid rise in rural populations has been reported [3]. There are no prospective studies that have identified risk factors of importance. The case-control INTERHEART study reported that standard risk factors such as smoking, abnormal lipids, hypertension, diabetes, high waist-hip ratio, sedentary lifestyle, psychosocial stress, and lack of consumption of fruits and vegetables explained more than 90% of acute CHD events in South Asians [14]. Similar conclusions were reached using urban-rural comparisons in risk factors and smaller case-control studies [3]. The INTERSTROKE study reported that ten common risk factors explained more than 90% incident haemorrhagic and thrombotic strokes [15]. The risk factors are similar to the INTERHEART study but the population attributable risks are different with greater importance of hypertension and lesser importance of diabetes and lipids. Reviews of epidemiological studies suggest that all the major risk factors are increasing in India [16-20]. Tobacco production and consumption has increased [16]. Smoking is increasing among young subjects (20-35 years) according to second and third National Family Health Surveys [17]. Prevalence of hypertension has increased in both urban and rural subjects and presently is 25-40% in urban and 10-15% among rural adults [18]. Lipids levels are increasing and serial studies from a north Indian city reported increasing mean levels of total, LDL and non-HDL cholesterol and triglycerides and decreasing HDL cholesterol [19]. Although there are large regional variations in prevalence of diabetes it has more than quadrupled in the last 20 years from <1-3% to 10-15% in urban and 3-5% in rural areas [20]. Studies have reported increasing obesity as well as truncal obesity, due to sedentary lifestyles and psychosocial stress in the country [21,22].

## Prevention of cardiovascular diseases

Control of risk factors has led to 50-80% decline in incidence of CVD in high income countries [5,23]. On the other hand, in absence of proper preventive approaches the risk factors are increasing in low and middle income countries [1]. WHO has classified prevention as population-based primordial, individual-based primary and patient-based secondary prevention [5]. Of the two approaches to prevention, the population approach is used to address the behavioural risk factors at the community level and its success depends on surveillance, population-wide education, partnerships with community organizations, assurance of health services, environmental change and policy and legislative initiatives. This approach addresses a selected list of modifiable risk factors such as diet, smoking and tobacco use, sedentary lifestyle, and availability of screening and diagnostic services. The high risk individual-based prevention approach should assess risk factors to determine individual risk. Medical interventions are often required. Secondary prevention strategies, on the other hand, comprise mainly medical interventions in addition to therapeutic lifestyle changes. A multi-factorial comprehensive approach that focuses on policy-based approach for prevention of cardiovascular disease in all parts of the world, especially in low and middle income countries, is suggested.

## Policy changes are effective

A large body of scientific evidence supports the concept that policy changes at the government level are the quickest way to improve population health including chronic diseases [4,23]. North Karelia, Finland was the first population level observatory where government-led policy changes (dietary fat control, smoking policies) coupled with population-based educational intervention reduced CVD mortality by 60-80% over the next twenty years [24]. Similar observations have been reported by countries in Europe, North America and Japan where the dietary and smoking policies were put into practice leading to reduced smoking and cholesterol levels [23]. A slightly different policy was adopted in the US where aggressive population-level risk factor control and pharmacotherapy by physicians led to better control of hypertension and hypercholesterolemia and reduction of CVD [23].

The decline in CVD mortality in high income European and North American countries has followed two phases [25]. The first phase in 1970-90s and was due to population based measures for risk factor control initiated by changes in policies on smoking, substitution of vegetable oils for animal fats and physical activity promotion. The second phase of decline from 1990's to date is ascribed to better management of risk factors and acute CVD syndromes and short-term as well as long term use of evidence based pharmacotherapies. Public healthcare financing and strengthening of primary, secondary and tertiary care is important [26]. Influence of policy changes on CVD mortality in different countries is summarised in Table 2[25-31]. It is observed that in countries where population based tobacco control policies, salt and fat control strategies and focussed control of multiple CVD risk factors (mainly hypertension and hypercholesterolemia) by physicians have been actively pursued there has been a significant decline in CVD incidence varying from 50-90% over a 20-30 year period. In middle income countries of Eastern Europe where such initiatives were delayed there has been a lesser decline (20-40%). On the other hand, in low income countries such as China and India no significant policy initiatives exist and in China there is evidence of increase in CVD mortality [31]. In India, reliable mortality data do not exist but there is evidence of increase in CVD prevalence over the same period [3].

**Table 2:** Policy changes in Europe, North America and other countries that led to decline in CVD mortality

Country	Political agenda		Risk factor prevention			Better risk factor and disease management				Decline in CVD mortality	
	**Strengthening of healthcare systems for acute and chronic CVD care**	**Public healthcare financing and insurance**	**Tobacco control policies**	**Food-modification initiatives**	**Physical activity promotion**	**Chronic diseases/CVD focused physician education**	**Aggressive population based pharmacological risk factor control**	**CVD focused primary care**	**CVD focused secondary/tertiary care**	**Period evaluated**	**Percent change**

Western Europe^23^	++++	++++	+++	++	++	++++	++	+++	+++	1970-2000	(-) 40-45%
Finland^22^	++++	++++	+++	++	+++	++++	++	+++	+++	1972-2007	(-) 75-80%
Germany^25^	++++	++++	+++	++	+++	++++	++	+++	+++	1980-2000	(-) 39-50%
Spain^22^	++++	++++	+++	+++	+++	++++	++	+++	+++	1970-2000	(-) 48-50%
England^26^	++++	++++	+++	++	++	++++	+++	++++	+++	1984-2004	(-) 48-52%
Australia^27^	++++	++++	+++	++	++	++++	+++	++++	+++	1968-2000	(-) 83%
USA^28^	+++	++	++	++	++	++++	++++	+++	++++	1970-2000	(-) 60%
Russia^23^	++	+++	++	+	+	++ +	++	++	+++	1970-2000	(-) 10%
Eastern Europe^23^	++	++	++	++	++	+++	++	++	++	1985-2000	(-) 16%
China^29^	+	+++	+	++	++	++	+	+	++	1985-2004	(+) 27-50%
India^3^	+	+	++	0	0	+	0	0	+++	No data	--

Scale of 0 to 4+.											

Thus, the decline is mortality from CVD in high income countries is due mainly to population wide decrease in risk factors, better risk factor management and control, and disease management strategies. Primordial prevention is focused as decreasing risk factor load in the population using strategies for increasing awareness and access through education regarding smoking and tobacco cessation, dietary modulation (low fat and high fruit and vegetables intake) and increased physical activity [26]. It also involves addressing the social determinants of health through improvement in daily living conditions, fair distribution of power, money and resources and continuous upgradation of knowledge, monitoring and skills [32]. Primary prevention is directed towards control of CVD risk factors such as smoking, hypertension, high low density lipoprotein (LDL) cholesterol, low high density lipoprotein (HDL) cholesterol, metabolic syndrome and diabetes so that onset of manifest CVD is avoided or delayed [26]. Secondary prevention is use of lifestyle changes, risk factor control and pharmacological strategies in patients with established CVD (CHD, stroke and others) and tertiary prevention is use of advanced techniques such as coronary interventions and bypass surgery in addition to secondary prevention strategies in patients with established disease [26]. Role of policy makers is important at the levels of all prevention.

## Lessons for India

**Figure 1:**
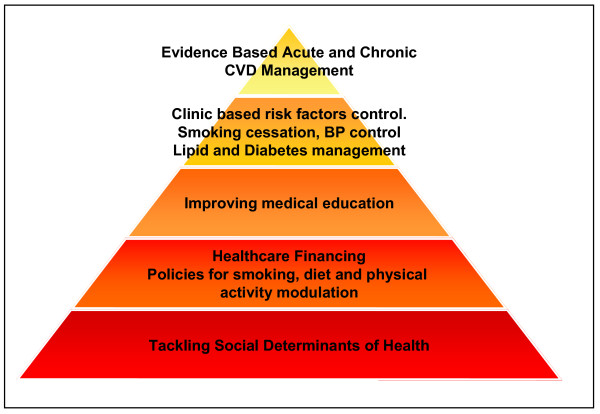
**Cardiovascular prevention pyramid**. The greatest benefit and CVD reduction is achieved by primordial prevention measures which involve tackling the social determinants, public health financing, population policies for smoking cessation, promotion of healthy diet and physical activity, and changes in medical education curriculum focussed on preventive care. Clinic based primary prevention strategies which involve control of blood pressure, lipids and diabetes are important. Care of acute CVD event and long-term CVD management with secondary and tertiary prevention therapies also contribute to reduction to CVD mortality.

The major challenge for the governments in low income countries such as India is to develop cost-effective strategies to respond to threat from CVD with aim to delay and eliminate premature onset and reduce mortality and morbidity [4,33]. Prevention and control from all types of CVD including CHD is a three pronged process and all these approaches are complimentary (Figure 1). Social determinants of health are very important for primordial, primary and secondary prevention and policies at the government level influence all.

### Primordial Prevention

These strategies are focussed on the population wide reduction of multiple cardiovascular risk factors. Rose developed the concept of CVD risk as a continuum in a population and showed that all pathophysiological factors were continuously distributed in a population [34]. He opined that high-risk and sick individuals simply represented the extreme end of the statistical distribution. From this hypothesis evolved the concept of population-wide control of risk factors. This would shift the mean levels of risk factors (e.g., cholesterol, systolic BP) in the standard normal curve to the left and decrease number of high-risk individuals. The principal concepts of the Rose hypotheses are continuity of risk and its population impact, more disease in subjects at small risk as compared to less numbers in people with high risk, prediction of cases using population mean of risk factors, difference in causes of CVD among population and individuals, societal characteristics that influence risk, and implications on policy, research and population action [34]. The policy and clinical actions for population actions for prevention are summarised below.

### Improving socioeconomic environment and literacy

**Figure 2:**
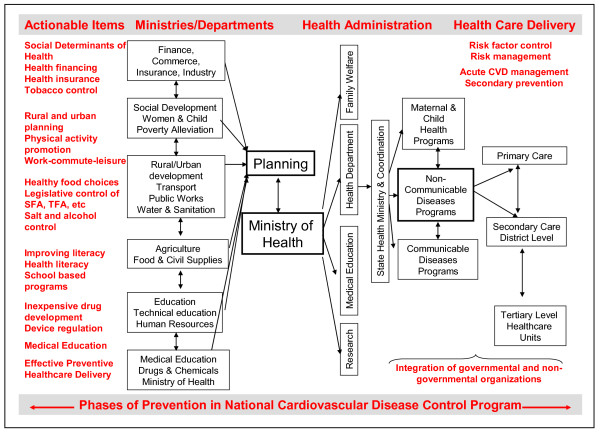
**Integration of various stake-holders for formulation of policies and implementation of cardiovascular disease prevention and control in India**. Ministry of planning should act as nodal point for action and coordinate and integrate activities of various ministries involved in planning, policy development and program implementation. Planning ministry along with ministry of health and its various departments should implement the national cardiovascular disease control program jointly with the state departments of health. There is a need to integrate various maternal and child health programs and communicable diseases programs with non-communicable diseases programs. Also required is a multi-level integration (horizontal and vertical) of various governmental and non-governmental organizations involved in healthcare delivery at the national and state level. SFA saturated fatty acids; TFA trans fatty acids; CVD cardiovascular disease

The social issues involved in occurrence of premature CVD and other non-communicable diseases are multiple and include the social gradient, stress, early life events, social exclusion, improper working conditions, lack of social support, addictions including tobacco/alcohol, food scarcity or excess and uneven distribution, lack of proper transport and illiteracy and low educational status [32]. There are macrolevel (governance failure, geopolitics, natural resources decline, economic policies, population growth, demographic trap, etc.) and microlevel (cultural barriers, poverty trap, lack of innovation and savings, absence of trade/business, technological reversal, adverse productivity shock, gender bias, adolescence-related, etc.) factors [30]. Multiple national programs exist in India to improve socioeconomic status of the population ranging from literacy improvement (National Literacy Mission and Right to Education Act) to employment generation (National Rural Employment Guarantee Act) and social security (Women's Health Plan). These policies are directed to the specific social issue or population group but not in context of disease prevention or control. Improving literacy reduces unhealthy behaviours (e.g., smoking) and increases awareness of risk factors [35]. It also promotes adherence to lifestyle and pharmacotherapies for primary and secondary prevention [36]. Both general literacy [37] and health literacy [38] should be part of the National Literacy Mission. Inter-ministerial collaboration is essential for policy implementation on CVD prevention and control (Figure 2) and the Indian National Commission of Macroeconomics and Health which encompasses ministries of health, finance, technical education, human development, youth affairs, sports, women and child development, agriculture, food and civil supplies, industry, commerce and transport is an important step forward [39].

### National CVD health programmes

The existing Indian national health programs are directed towards communicable diseases and maternal and child health [40]. Although national program for control of cardiovascular diseases and diabetes has been initiated as a pilot it has not been scaled due to paucity of funds [41]. We believe that it is a priority and the program and must be spread widely and scope and funding substantially increased. National Rural Health Mission of government of India [42] should focus on improvement of healthcare systems for non-communicable diseases and chronic care [43,44]. The National Urban Health Mission [45] which has been in a planning stage for a long time also needs to be urgently implemented. Clearly a political will to initiate and sustain these programs is needed. Acute cardiovascular events (acute myocardial infarction or stroke) should be deemed notifiable similar to certain infections [40].

### Healthcare financing and universal insurance

An important area for CVD control is healthcare financing by the government. In India the total healthcare spending is about 5% of the gross national product (GNP) but government spends less than 1% of GNP [4]. Treating CHD is expensive [46] but unfortunately all the focus of the government is on development of tertiary care facilities. It is possible to reduce the mortality and burden of CHD by population wide policies [4]. Financing is needed not only for implementation of programs for tobacco, alcohol and dietary fat control but also to improve urban design and provide better education within the existing national programmes. Health insurance for acute and chronic diseases is virtually non-existent in India [47]. More than 80% population pays out of pocket for health related expenses and every year almost 30 million individuals are pushed into poverty for catastrophic healthcare expenditure [48]. India has the most privatised healthcare system in the world (<20% public funding) compared to the US (the most privatised system) where the government health expenditure is about 50% of the total [49]. In GNP terms too, at 0.9 per cent of the governmental health care spending in India is one of the lowest in the world [49]. A few state governments have joined hands with private insurers to provide health-insurance to general population but the impact of such schemes has not been evaluated [50].

### Changing medical education curriculum

Primordial and primary prevention of chronic non-communicable diseases especially CVD and CHD are not major component of medical school curricula in India [40]. At policy level, Medical Council of India should implement this change. Continuing medical education for general physicians, general practitioners and others about advances in cardiovascular and other forms of therapy should be mandatory [51]. Physicians should be encouraged to use global risk-assessment tools such as the Framingham Risk Score [52]. They have to be aware of various CVD guidelines [53]. Although there is evidence in India that devoted clinicians manage their patients according to the guidelines [54], the uptake of preventive efforts is limited [46]. Establishment of systems to address the multi-level contexts that influence the development and maintenance of prevention-related health behaviours is important [33,55]. Hospitals and health care systems should be encouraged to develop and provide preventive cardiology services and systems for the community [51]. And finally there should be creation and certification for preventive CHD specialists who can train the practitioners and paramedics in strategies of prevention and health promotion. Training of healthcare workers and allied health professionals for systematic or opportunistic screening is important [26,51,53]. This would lead to early diagnosis of risk factors and their management. There is also a need for creation of cadre of non-communicable disease health care workers for not only screening but also for ensuring monitoring of individuals and ensuring compliance. These workers should also be trained to educate the populace in early diagnosis of acute CVD events and ensuring referral.

### Tobacco control

An important policy action for CVD prevention and control is enforcement of strict anti-tobacco laws[6]. It is now well recognised that smoking in any form (cigarettes, bidis and others) as well as use of non-smoked tobacco is equally dangerous to health, especially CVD health [56]. Smoking and tobacco use is rampant among the rural subjects and in low socioeconomic status urban subjects [35]. The comprehensive legislation on tobacco control and adoption of the WHO Framework Convention on Tobacco Control (FCTC) by government in India is an important step in this direction [57]. Studies from Europe and North America have reported that strict enforcement of FCTC have resulted in reduced admissions due to CHD and decreased mortality [58]. Indian laws need to be amended to comply with the provisions of the FCTC [59]. There is a considerable gap in policy and its application. According to a survey the enforcement of FCTC in India is less than 20% [60]. The government needs to approve anti-smoking laws and educate the public through organized programs with media support and enforce the tobacco control legislation strictly.

### Healthy diet, salt and alcohol control

Diet is a very controversial area and the current evidence based practices adopted by the western countries should be carefully evaluated and cautiously implemented [32,33]. WHO has concluded that as a society moves up the socioeconomic scale there are substantial changes that occur in diet [61]. These include greater consumption of calories, fats, saturated fats, trans-fats, salt, refined carbohydrates and sugars and decreased intake of high-fibre foods, legumes and vegetables. A healthy diet should be the opposite. Guidelines promoted by WHO [61] and Indian national committees [33,62] suggest: (i) increased intake of fruits and vegetables (at least 500 g per day), legumes and whole grain foods; (ii) reduction of the intake of fried foods, processed foods, soft drinks containing calories, and other unhealthy foods; (iii) limitation on the daily intake of total fat to 25-35% of calories, and saturated fat <7% of the calories, by limiting the use of butter, ghee, full-fat dairy products, trans-fats, and tropical oils (palm oil and coconut oil) and increased intake of mono-unsaturated fats up to 20%; (iv) reduction of the dietary glycemic load by cutting down on the carbohydrates, especially refined carbohydrates; (v) reduction of the intake of salt to <2.5 gm sodium or <6 gm of salt (one teaspoon of salt) per day; and (vi) moderation in the use of nuts, lean meat and fish and alcohol. Government policies are important to translate knowledge into practice [63]. For years the food and nutrition policies of the Indian government have focused on problem of undernutrition [39]. The dual epidemic of under- and over-nutrition needs a different response. Focus should be on a balanced healthy diet that tackles problems of both rather than on overweight/obesity alone. India is, also, unique because more than 90% of consumed food is home-made. Following steps can have major immediate effects: (i) promotion of healthy nutrition in the general population by implementation of various government of India guidelines on chronic diseases, diabetes and obesity; (ii) creation of culturally sensitive health educational material; (iii) subsidies for producing and distributing healthy foods and fruits; (iv) penal taxation on unhealthy saturated and hydrogenated fats and subsidies on vegetable oils; (v) food labelling including portion size, calories, total fat, saturated fat, trans-fat, protein, carbohydrates, sodium, sugar and fibre; (vi) healthy foods availability at schools and work sites; and (vii) reduction of salt content in the processed food.

Salt is important in genesis and perpetuation of hypertension and has a direct correlation with CVD [64]. Indian diet has a very high salt content, typically 10-12 g of salt intake per day as recommended by government agencies [65]. A lower intake is (5-6 g salt/day) suggested by international guidelines [26]. Recommendations regarding alcohol intake for CVD prevention are confusing [66]. The overall harm of alcohol due to high blood pressure, liver disease and accidents and injuries and is much more than protective effects on CVD [67] and as policy alcohol should be discouraged.

### Policies for physical activity

Regular physical activity is extremely important for CVD prevention [68]. The recommended physical activity is 30-to-45 minutes of moderate-intensity activity such as brisk-walking every day. In India, work-related physical activity is confined to rural and urban low socioeconomic status subjects [69], while leisure time physical activity is limited to a motivated few in urban areas [70]. Evidence from Europe and North America reports beneficial effect on population based initiatives on increasing physical activity with a public health perspective [71]. Occupational physical activity and creating separate time for employees has been the focus of international agencies [72]. There are examples of multiple comprehensive worksite health promotion programs that focus on improvement of physical activity [73,74]. In India, an industrial worksite intervention program reported significant change in health related behaviours with reduced tobacco use and increased physical activity [75]. Habit of active participation in physical activity programs, games and sports begins in early childhood and track to adulthood, therefore, physical education should be given greater emphasis in schools and colleges [76]. Yoga can be part of the physical activity among the school children as its regular participation not only improves physical capacity but also improves adherence to other strategies for improving health [33]. In a study in India 40000 school children in were provided educational intervention for a 6 month period which resulted in improved health literacy and health behaviours at one year [77]. Policy changes have to be implemented and it is heartening to note that national and state-level educational boards have included chronic disease prevention in the course curriculum.

Urban planning is important for public health and can improve human well-being, emphasize needs assessment and service delivery, manage complex social systems, focus at the population level, and rely on community-based participatory methods [72,78]. The current development of cities has been haphazard and most are conglomeration of houses and narrow lanes with no place to walk safely [79]. For CVD prevention there should be encouragement of construction and use of foot-paths and bicycle-paths. A uniform urban development policy is needed which should not only be guided by aesthetics but also by the health needs of the population. Traditionally, these plans include planning for community needs in transportation, housing, commercial/office buildings, natural resource utilization, environmental protection, and health-care infrastructure [78].

### Primary and secondary prevention

This strategy focuses on control of risk factors [26]. Although a large number of CVD risk are described interventions focussed on the major risk factors (smoking, high BP, high LDL cholesterol, low HDL cholesterol and diabetes) have shown to lead to substantial decline in incidence of CVD [80]. Primary prevention strategy should focus on improving healthcare delivery and target-oriented control of risk factors [68].

### Healthcare delivery for primary prevention

In India the healthcare delivery mechanisms are very chaotic [4]. For proper delivery of chronic health care the medical infrastructure needs to be structured like a pyramid [45]. In this pyramid the maximum emphasis has to be at the primary level, i.e. at the level of first contact that should be easily accessible (both physically and in terms of affordability) by everyone. Each successive rung needs to be equipped to perform more complex tasks for more complex conditions. This has to be followed up with a referral system along this pyramid [51]. Such a system minimises waste (viz. in the form of trivial or self-limiting illnesses being treated at district or state level hospitals) and enhances accessibility. In India on paper we have such a system in operation [45] but in reality this system is woefully inadequate. The infrastructure below the district level is rudimentary. This places enormous burden on facilities at state and district levels. The reason for this is two-fold. First, while in a pyramid the strength of the base is most important, we somehow have this erroneous notion that the excellence of the system depends on the apex, i.e., specialty hospitals in metropolitan centres. As a policy, preventive healthcare and chronic disease surveillance should be delivered by primary care physicians or trained community health workers [81]. As a large proportion of population in India receives healthcare from non-governmental sector [82], at a policy level there should be an effective integration of government and non-government agencies involved in delivery of primary health care [83] (Figure 2).

### Risk factor control

Aggressive primary prevention is critical to preventing CVD in those who are in high-risk category with the four key objectives: (i) avoidance of all tobacco products and passive exposure to tobacco, (ii) control of dyslipidemia, (ii) control of blood pressure, and (iii) control of blood glucose and diabetes through intense lifestyle modification and medications if necessary [1,5,53]. Community worker based model for chronic disease risk factor control has been tried in many settings with variable results [81]. This strategy is useful in management of infections, pregnancy related outcomes and malnutrition. Trials are underway in India to assess their importance in chronic diseases. Use of practitioners of alternative systems of medicine could be important for chronic diseases risk factor management [83]. India has a strong tradition of religiosity and all *gurus *recommend prudent lifestyle- with focus on smoking cessation, balanced diet, physical exercise and mental relaxation- important for CVD control.

### Secondary prevention

Acute cardiovascular disease management and appropriate long term medical care is crucial in preventing CVD related mortality [84]. A substantial reduction in CVD related mortality in high income countries is due to better acute coronary care and long term management [25]. On the other hand availability of care and its standard is variable in low income countries [1]. There is need to develop better quality CVD care systems and manpower in these countries.

There is a need to develop publicly funded primary and secondary healthcare systems for acute CVD care [51]. Acute coronary events should be made a notifiable condition. In India, management practices in acute coronary syndromes are guidelines driven and optimal [54]. Guidelines recommend that lifestyle changes recommended above, aspirin, beta-blockers, angiotensin converting enzyme (ACE) inhibitors and statins be used in all patients with CVD [84]. There was low use of secondary prevention therapies in Europe and North America in late 1990's but the situation has improved due to policy focus on better management [85]. In India there is low use of various evidence based medicines, especially statins and beta-blockers and a rapid decline in use as the patients move from tertiary to secondary and primary care [86]. This is due to multiple barriers to proper management at healthcare systems, healthcare providers and patient level. Health systems related barriers are in the realm of government policy and action and need major efforts.

## Implementing policy interventions

Healthcare policy implementation requires partnerships between multiple stakeholders. This is also true for CVD prevention and control [4,44]. National health programs in low income countries are often managed as vertically integrated systems of care with no horizontal interaction. For example in India there are more than two dozen national programmes for control of communicable and non-communicable diseases and maternal and child health [40]. There is very little integration at the grass-roots level that leads to low quality expensive care. Figure 2 shows the proposed flowchart of horizontal and vertical integration of various stake-holders involved in CVD prevention. The fundamental importance of social determinants of health in primordial, primary and secondary CVD prevention is highlighted.

At the ministerial level it has to be realised that there are multiple determinants of CVD health and all these need focus. There is need for integrated approach to policy making with participation of ministries of health, finance, commerce, industry, social development, rural and urban development, transport, agriculture, food and civil supplies, education, human development, information and broadcasting, telecommunications, drugs and chemicals and some others. Planning ministry should act as a nodal point for all activities (Figure 2). An apex committee to integrate activities of various ministries should be formed. Priority agenda for this committee should be to formulate guidelines for implementing agenda to modify the social determinants of health, effective implementation of national CVD control programme, change the medical education curriculum with focus on chronic diseases and improving public health financing and develop low-cost insurance schemes. Regular assessments of the progress and external audits to evaluate performance should be performed. There should also be a good coordination between the central and various state governments. Although there is need for good secondary care, strengthening of primary health care systems to take care of chronic diseases with facilities for risk factor identification, risk factor control and secondary prevention therapies is essential as suggested by WHO and other organizations [87]. With proper management of health systems using modern technologies it should be possible to integrate various programs, both horizontally and vertically, and provide better acute and chronic diseases control [88]. All this requires political will and unless such a desire exists in the country the march of CVD shall continue leading to premature mortality especially among the low socioeconomic populations [89].

## Conclusions

The epidemic of CVD in India needs an urgent policy response for its control. It is no longer the disease of the rich or the well-educated. Strategies that have been successful in upper-middle income countries are available and need to be implemented [25]. Most of the achievements are attributable to social change involving culture, housing and food [90]. Table 3 and Figure 2 prioritize the agenda for the government. Political and bureaucratic will and policies for improving human development index should be implemented. Initiation of national cardiovascular diseases control program is important. The medical curricula need to be extensively revised to incorporate these diseases into the mainstream. Financing mechanisms have to be developed for supporting healthcare infrastructure at primary and secondary levels and for public-funded health insurance. Suitable policies for tobacco control, dietary fat control and better physical activity need to be enforced. Both primary prevention for target oriented control of CVD risk factors and pharmacotherapy based secondary prevention need to be implemented. Facilities for management of CVD risk factors in primary care and acute and chronic disease management in secondary care are needed. Implementation of these policies can delay the occurrence of acute cardiovascular event by at least 10 years, reduce the mortality burden by 25-30%, decrease utilization of tertiary care, save money, and reduce the inexorable march of cardiovascular diseases in India.

**Table 3:** Policy agenda for CVD control

Policy domain	Existing policies or programs in India	Unmet actionable needs
Socioeconomic and education	National literacy mission, right to education act	Strengthen policy initiatives
	National rural employment guarantee act	Linking these to health
		Inter-ministerial collaboration
National CVD control program	Pilot phase of national CVD and diabetes control program	Scaling up and integration with NRHM and NUHM
	National health programs (NRHM, NUHM)	
Healthcare financing	State level initiatives for families designated below poverty line	Health insurance for CVD including for risk factor management, acute care and secondary prevention
	Multiple public and private insurance providers	
		Integration and social marketing of existing initiatives
Medical education and training of healthcare workers	Largely profession driven, cure-centric continuing medical education events	Structured, public-health, preventive approach
		A formal preventive cardiology education and certifications
Tobacco control	India is a signatory to FCTC and has tobacco control legislations in place	Strengthen implementation of FCTC guidelines and legislations
Healthy diet	Minimal organized efforts	Focus on control of saturated fats, trans fats, salt and alcohol
		Industry initiatives for alternate strategies
Improved physical activity	Minimal organized efforts	Better urban planning with inter-ministerial collaboration
		Worksite and school based interventions
Aggressive primary prevention and preventive healthcare delivery	Existing network of primary health centres, district hospitals, and teaching hospitals in public sector	Needs orientation to CVD and diabetes care
	A larger number of private care providers, mostly unorganized and a smaller more organized corporate sector in urban areas	Needs quality control and standardization

Evidence based acute care and secondary prevention	Minimal and fractured	Better acute care
		Chronic care delivery improvement and use of evidence based therapies

CVD cardiovascular diseases; NRHM National rural health mission; NUHM National urban health mission; FCTC Framework convention on tobacco control

## Conflicts of interests

All the authors are members of Indian Cardiovascular Research and Advocacy Group at St John's Research Institute, Bangalore and financially supported by centre for excellence grant (BAA HV0912 650066553) from National Heart Lung and Blood Institute under the Global Health Initiative of National Institutes of Health, USA. There are no other financial conflicts of interest relevant to this article.

## Authors' contributions

RG developed the theme of the article, wrote the first draft and was involved in all subsequent revisions. SG reviewed the first and subsequent drafts and was involved in all the major revisions. RJ reviewed the draft versions of the article, contributed to the tables and figures and provided important comments. DX reviewed the manuscript and provided important comments. All authors read and approved the final manuscript.
